# Linkages between soil organic carbon fractions and carbon-hydrolyzing enzyme activities across riparian zones in the Three Gorges of China

**DOI:** 10.1038/s41598-020-65200-z

**Published:** 2020-05-21

**Authors:** Dandan Zhang, Junjun Wu, Fan Yang, Qiong Chen, Jiao Feng, Qianxi Li, Qian Zhang, Weibo Wang, Xiaoli Cheng

**Affiliations:** 1 Key Laboratory of Aquatic Botany and Watershed Ecology, Wuhan Botanical Garden, Chinese Academy of Sciences (CAS), Wuhan, 430074 P.R. China; 2 Graduate University of Chinese Academy of Sciences, Beijing, 100049 China; 3 School of Ecology and Environmental Sciences, Yunnan University, Kunming, 650091 China

**Keywords:** Carbon cycle, Riparian ecology, Wetlands ecology

## Abstract

The effect of flooding on soil enzyme activities and soil organic carbon (SOC) dynamics remains a widely debated topic. Here, we investigated spatial variations in C-hydrolyzing enzyme activities, soil C contents in different fractions [i.e. labile and recalcitrant carbon (LC and RC)] from 6 sites with four different elevations at two soil depths (0–10 cm and 10–30 cm) in riparian zones of the Three Gorges Reservoir, China. At region scales, the SOC, RC contents, and RC/SOC (RIC) generally showed decreasing tendency from the upstream to the downstream. The C-hydrolyzing enzyme activities were higher in the midstream compared to other sites, which did not correspond well with the changing trend of SOC content, but matched with the spatial variation in LC content. At ecosystem scales, the RC and RIC declined with decreased elevations, but the LC showed opposite trend. Whereas, the four C-hydrolyzing enzyme activities and the specific enzyme activities were corresponded well with the changing trend of LC content. Soil C contents and enzyme activities were generally higher in top soil than deep soil across sites and elevation zones. These results reveal that the LC is the tightest factor in regulating C-hydrolyzing enzyme activities, whereas the soil C quality (i.e. RIC) and flooding collectively drive C-hydrolyzing enzyme activities possibly by affecting decomposition rates of SOC in the riparian zones.

## Introduction

Anthropogenic activities such as large hydroelectric project can directly affect ecosystem processes and function by altering vegetation types, soil properties and hydrology^1,2^, and ultimately modify soil carbon (C) and nitrogen (N) cycling^3–5^. Soil organic carbon (SOC), one of the largest and most susceptible C pools of the earth surface ecosystem, can act either as source or sink for C primarily counting on multiple factors including human activities^6–10^. It is well known that the SOC pool is generally determined by the balance between litter C input and decomposition^7,11^, while the SOC decomposition process is predominantly catalyzed by a variety of extracellular enzymes^12–14^. Particularly, these enzymes can hydrolyze substrates and catalyze important transformations involving in soil organic matter (SOM) synthesis^15,16^. Although some studies have evaluated changes in soil C and N dynamics under large hydroelectric project^3,4,17^, how spatial variations in SOC dynamics link to C-hydrolyzing enzyme activities at region and/or ecosystem scales under large hydroelectric project remains largely unclear.

The linkages between SOC and C-hydrolyzing enzyme activities can be influenced primarily by altering vegetation types under large hydroelectric projects^2,3,18,19^. Most SOC exclusively originates from plant residues on site, and hence alterations in vegetation type are expected to change the quality and quantity of SOC, which in turn can affect C-hydrolyzing enzyme activities^20–23^. For instance, previous studies have reported that soil hydrolytic enzyme activities are tightly related to SOC content^12,23,24^, particularly, the labile C (LC) derived from fresh litter input has been generally regarded as the major source of energy for microbes producing hydrolytic enzymes^25,26^. In contrast, the fraction of recalcitrant C (RC) such as lignin and fatty acids is hardly utilized by microbes for producing hydrolytic enzymes, which can hinder microbial decomposition and hence promote C sequestration ^25,27^. Meanwhile, enzyme production secreted by microbes also relies on soil N availability, changes in soil C: N ratios can greatly impact soil enzyme activities^28^. It has been well reported that the SOM with low C: N ratios can induce microbial demand for C, and thus could produce enzymes to hydrolyze SOM for available C^24,29^. On the other hands, extracellular enzymes could be stabilized by SOC via the formation of enzyme-humus complexes, leading to change in enzyme activities^30,31^, thus, changes in enzyme activities would in turn reflect modifications in SOC stocks. In addition, several studies found that enzyme activities usually decreased with depth, possibly reflecting a significant proportion of metabolic transformations when unfavorable environmental conditions inhibited microbial growth^32^. However, the relationship between SOC dynamics and C-hydrolyzing enzyme activities are inconsistent in various studies, possibly due to related multiple controls, especially when soil conditions are affected by hydrologic pulsing under large hydroelectric project area.

It is widely recognized that hydrology under large hydroelectric project exert significant impacts on soil C dynamics and C-hydrolyzing enzyme activities^5,29^. Long-term water submergence usually results in observable changes in vegetation types and SOM decomposition, because soil aerobic surface layers can largely convert to anaerobic conditions^2,33^. The frequency of hydrological pulsing with carrying C-rich sediment usually leads to increase in soil C pools^5,34^. Water submergence can also reduce the input of fresh OC from plant litter and root exudates because of less oxygen availability for plant roots^35^. Meanwhile, enzyme activities could be indirectly affected by soil pH and microclimates under large hydroelectric project^1,5,36^. For instance, Amendola *et al*.^5^ have found that increase in soil moisture results in higher enzyme activities, with soil becoming dry condition, the substrate diffusion and oxygen content can limit enzyme activities. However, Guenet *et al*.^37^ have demonstrated that some enzyme activities are the highest in the soil with intermediate moisture, followed by moist soil and then dry soil. Additionally, increased soil temperatures with changes in oxygen availability are expected to have a substantial impact on the enzyme activities in wetlands^29^.

Since the completion of the Three Gorges Dam in 2008, a total area of 350 km^2^ water level fluctuation zone has been formed in the Three Gorges Reservoir with water level fluctuation from 145 m above sea level in summer (May to September) to 175 m in winter (October to April)^1,38^. The reversal of submergence time and prolonged inundation dramatically lead to change in soil moisture, pH and vegetation types^32,34^, and these changes ultimately affect soil C cycling and enzyme activities. Previous studies have investigated the spatial distribution of plants^18^ and soil N dynamics^4^, but little is known about spatial variations in SOC fractions and enzyme activities in water level fluctuation zone in the Three Gorges Reservoir. To address this major gap, the objective of this study is to reveal the linkage between spatial variations in SOC dynamics and C-hydrolyzing enzyme activities (α-glucosidase, AG; β-glucosidase, BG; 1,4-β-cellobiosidase, CB; 1,4-β-xylosidase, XS) effected by water flooding in Three Gorges. Specifically, we hypothesized that 1) the SOC contents and fraction (LC, RC, and RC/SOC) would significantly differ among sites and elevations, possibly due to changes in vegetation types induced by water flooding. 2) whether the C-hydrolyzing enzyme activities would be tightly related to soil C quality (i.e. RC/SOC) and fraction in the riparian zones.

## Results

### Plant traits, soil properties

Plant aboveground biomass, plant C and N generally were higher in the upstream sites (CS, FL, and ZX) compared to the downstream sites (WS and BD), while the C: N ratios showed opposite trend (Table 1). In contrast, plant aboveground biomass, plant C and N were significantly higher in high and middle elevations compared to the control, with no significant difference between high and middle elevations. There was no significant difference in plant C: N ratios among elevations. Similarly, soil pH and moisture increased from the control to low elevation zone, while soil temperature showed opposite trend (Table 2). There were no significant differences in soil pH, moisture and temperature between soil depths (Tables 2 and 3).

**Table 1:** Plant traits under different sites and elevations (see Fig. 1) in the water level fluctuation zone of the Three Gorges Reservoir, China.

		Biomass (g/m^2^)	Plant C (g/m^2^)	Plant N (g/m^2^)	Plant C: N ratios
Sites (S)	CS	678.06 ± 160.62	230.89 ± 54.93	10.24 ± 2.20	23.18 ± 3.19
	FL	527.04 ± 94.84	177.28 ± 30.18	11.34 ± 2.39	17.58 ± 2.60
	ZX	695.02 ± 77.02	252.99 ± 26.94	8.80 ± 1.97	37.55 ± 6.19
	WZ	364.95 ± 35.31	134.17 ± 13.87	6.00 ± 1.01	24.46 ± 2.43
	BD	362.78 ± 22.18	128.88 ± 9.65	5.47 ± 0.54	25.19 ± 2.70
	ZG	512.58 ± 89.52	185.66 ± 30.79	6.23 ± 0.69	30.81 ± 4.17
Elevations (E)	C	322.73 ± 33.63	117.58 ± 13.52	5.59 ± 0.67	24.85 ± 4.02
	H	648.74 ± 80.43	230.07 ± 27.38	9.19 ± 1.36	27.82 ± 2.03
	M	598.74 ± 61.00	207.28 ± 20.13	9.25 ± 1.38	26.71 ± 2.58
Source of variation	S	7.33***	7.76***	4.42**	8.514***
	E	21.66***	21.92***	6.42**	NS
	S × E	6.25***	6.61***	4.06***	8.557***

Note: CS, Changshou; FL, Fuling; ZX, Zhongxian; WZ, Wanzhou; BD, Badong; ZG, Zigui; C, control, elevation>175 m; H, high elevation between 160–175 m; M middle elevation between 145–160 m. Data are expressed as mean ± SE (site: n = 9, elevation: n = 18). Plant C, plant carbon content; Plant N, plant nitrogen content; S, sites; E, elevations. Significance effects of the sites and elevations on plant traits were based on Two-way ANOVA (NS = not significant; *P < 0.05; **P < 0.01; ***P < 0.001; numbers are F-values).

**Table 2:** Soil properties under different sites, elevations and depths.

		pH	SM (%)	ST (°C)
Sites	CS	7.98 ± 0.05	33.86 ± 2.18	29.73 ± 0.62
	FL	7.96 ± 0.12	25.75 ± 2.19	29.72 ± 0.47
	ZX	7.75 ± 0.16	26.62 ± 2.28	30.59 ± 0.73
	WZ	7.34 ± 0.19	24.03 ± 2.55	29.32 ± 0.66
	BD	8.01 ± 0.05	19.28 ± 1.13	27.03 ± 0.40
	ZG	6.60 ± 0.17	25.52 ± 1.49	26.26 ± 0.48
Elevations	C	6.93 ± 0.16	18.53 ± 0.89	32.38 ± 0.40
	H	7.56 ± 0.13	20.32 ± 1.28	29.80 ± 0.34
	M	7.80 ± 0.11	27.79 ± 1.65	27.49 ± 0.26
	L	8.14 ± 0.03	36.73 ± 1.38	25.43 ± 0.18
Depths (cm)	0–10	7.55 ± 0.10	26.44 ± 1.31	28.77 ± 0.38
	10–30	7.66 ± 0.09	25.24 ± 1.21	28.77 ± 0.38

Note: Data are expressed as mean ± SE (site: n = 24, elevation: n = 36, depth: n = 72). L, low elevation <145 m; SM, soil moisture; ST, soil temperature. See Tables 1 and 2 for the abbreviations.

**Table 3:** Statistical significance of the effects of sites, elevations, depths and their interactions on soil enzyme activities and soil properties based on Multiple ANOVA (NS = not significant; *P < 0.05; **P < 0.01; ***P < 0.001; numbers are F-values).

Parameter	Sites(S)	Elevations (E)	Depths (D)	S × E	S × D	E × D	S × E × D
AG	66.10***	17.40***	28.86***	14.10***	13.40***	3.24*	7.17***
BG	67.04***	25.95***	27.68***	13.63***	10.65***	6.44**	7.87***
CB	33.63***	20.45***	53.53***	11.45***	15.91***	7.89***	8.79***
XS	58.37***	29.32***	59.10***	14.78***	11.51***	5.29**	8.60***
EA/SOC	40.49***	32.03***	NS	10.72***	6.25***	8.72***	9.7***
SOC	142.61***	42.83***	219.18***	8.26***	8.27***	13.11***	5.34***
C: N ratio	42.89***	NS	NS	3.93***	NS	NS	NS
LC	19.17***	41.50***	81.36***	9.50***	9.30***	9.52***	6.47***
RC	146.34***	79.55***	125.27***	12.07***	3.51***	6.03**	3.03**
RIC	30.06***	74.77***	NS	13.15***	5.19***	NS	1.9*
pH	99.67***	126.71***	NS	19.96***	NS	NS	NS
SM	22.25***	102.91***	NS	7.97***	NS	NS	1.84*
ST	337.43***	1533.73***	NS	31.03***	NS	NS	NS

Note: AG, α-glucosidase; BG, β-Glucosidase; CB, 1,4-β-cellobiosidase; XS, 1,4-β-xylosidase; EA/SOC, enzyme activity per unit soil organic carbon; SOC, soil organic carbon; LC, soil labile carbon; RC, soil recalcitrant carbon; RIC, RC/SOC; SM, soil moisture; ST, soil temperature.

### Soil organic C, labile and RC contents

**Figure 1:**
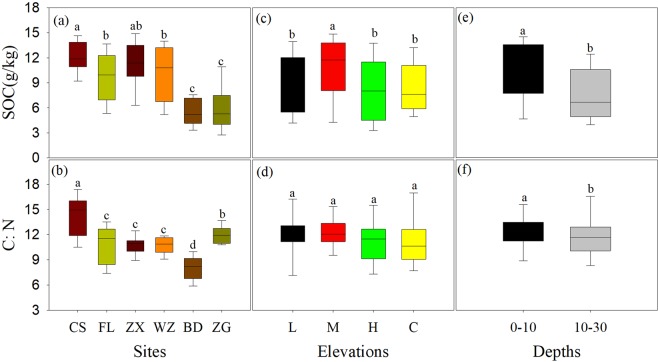
Soil organic carbon (SOC) and soil C: N ratio under different sites (**a**,**b**), elevations (**c**,**d**) and soil depths (**e**,**f**). Different lowercase letters over the bars indicate statistically significant differences among sites, elevations and depths, respectively, at P < 0.05 based on least significant difference (LSD) analysis in One-Way ANOVA. See Table 1 for the abbreviations.

**Figure 2:**
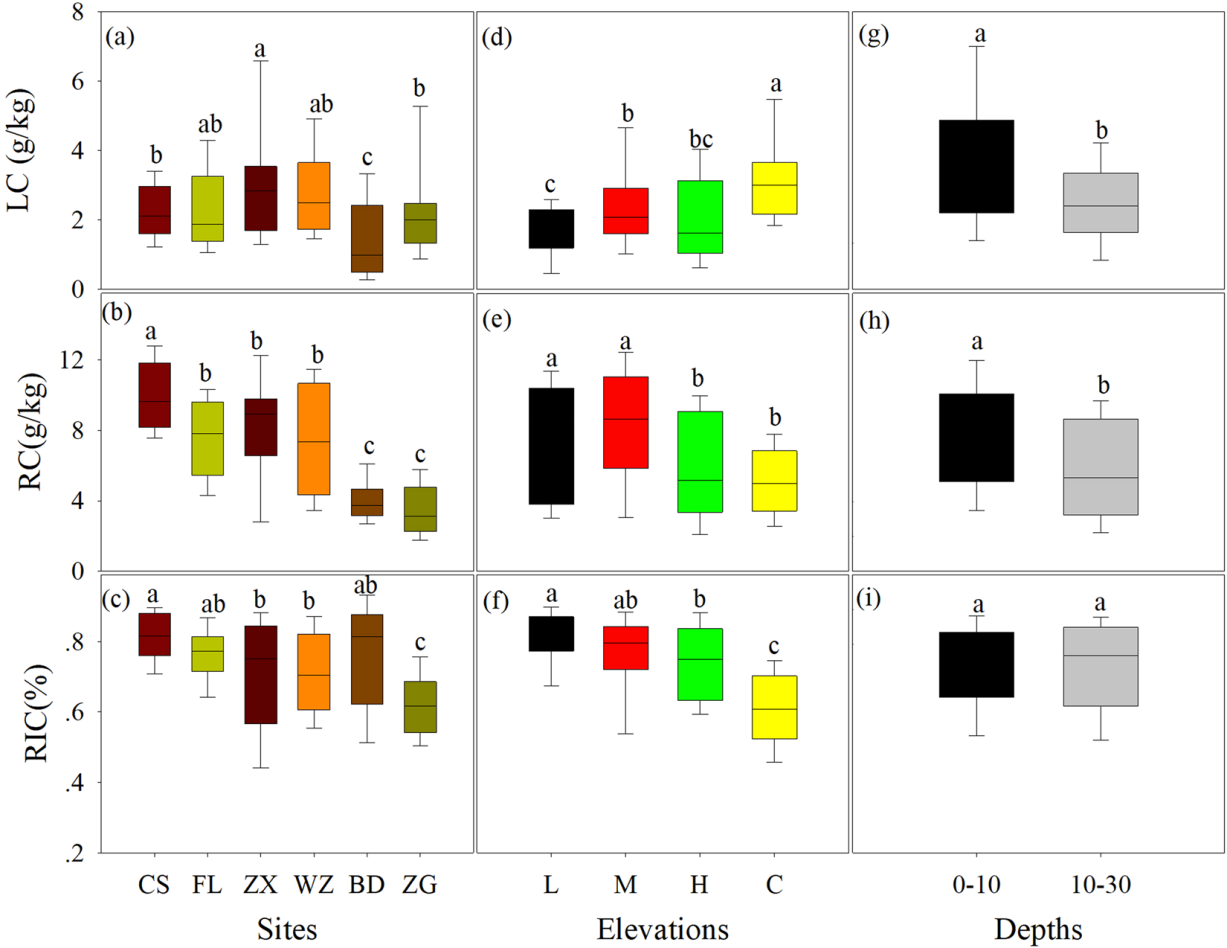
Soil labile carbon (LC), soil recalcitrant carbon (RC) and recalcitrance index for C (RIC) under different sites (**a**–**c**), elevations(**d**–**f**) and soil depths (**g**–**i**). See Table 1 and Fig. 2 for the abbreviations and explanations.

Among sites, SOC, C: N ratio of organic matter pool, RC, and RIC generally decreased from the upstream to the downstream, the higher LC content was observed in ZX than other sites (Figs. 1a and 2a–c). Among elevations, SOC content was higher in the middle elevation than other elevations (Fig. 1c), while C: N ratio of organic pool did not significantly change between different elevation zones (Fig. 1d). In contrast, RC and RIC declined from the high elevation to low elevation zone, whereas the LC showed opposite trend (Fig. 2d–f). In general, SOC, LC, RC and C: N ratios were higher in top soil than deep soil, but RIC did not significantly change between two soil layers (Figs. 1e,f and 2g,h)

### Soil enzyme activities

**Figure 3:**
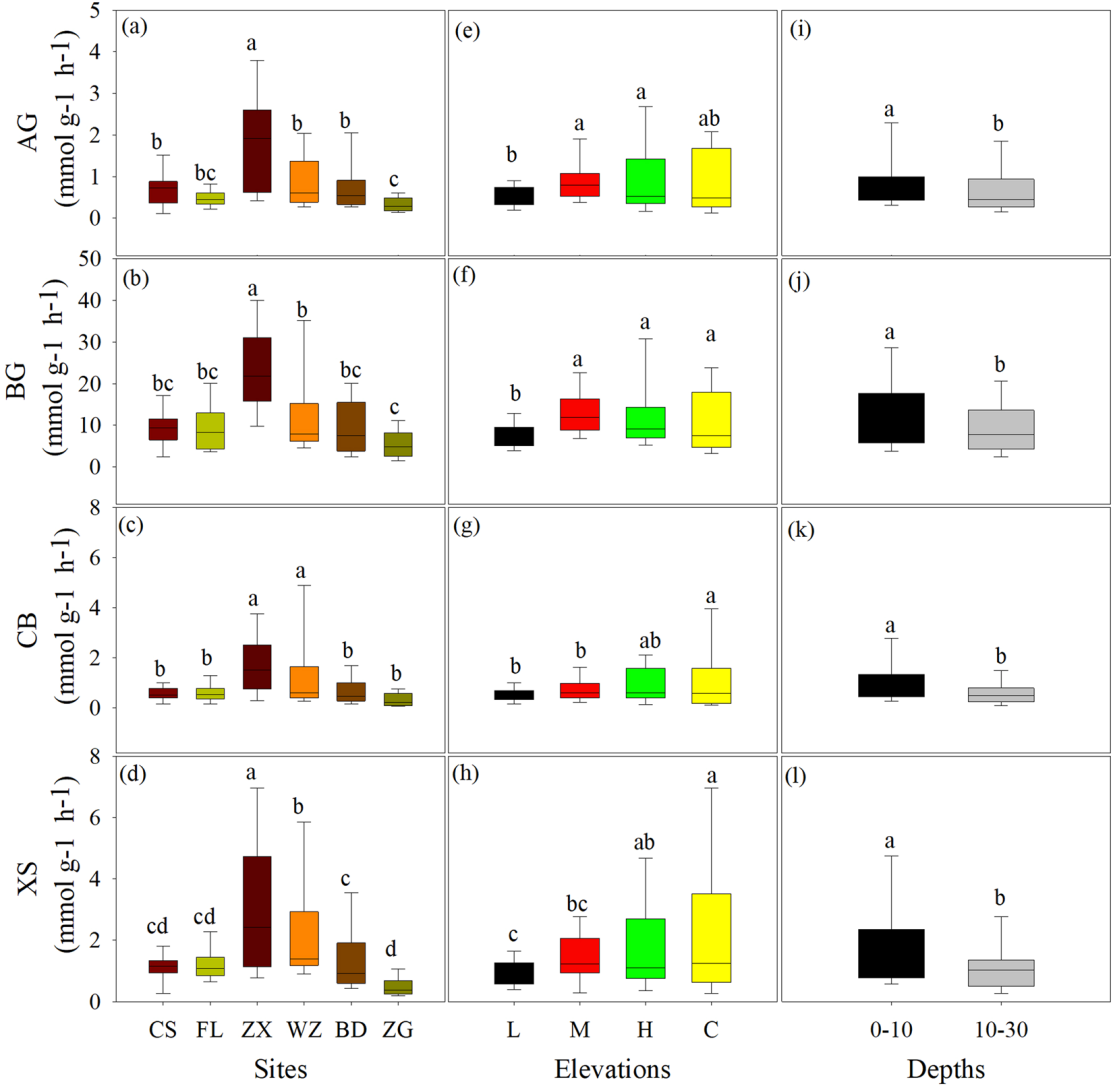
C-acquiring hydrolytic enzyme activities under different sites (**a**–**d**), elevations (**e**–**h**) and soil depths (**i**–**l**). See Table 1 and Fig. 2 for the abbreviations and explanations.

**Figure 4:**
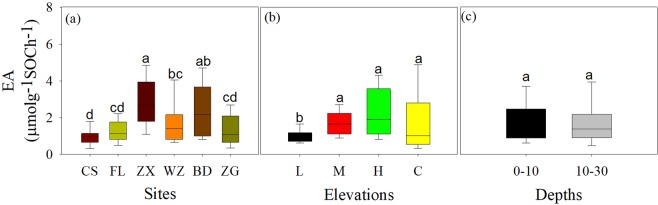
Enzyme activity per unit soil C (EA/SOC) under different sites (**a**), elevations (**b**) and soil depths (**c**). See Table 1 and Fig. 2 for the abbreviations and explanations.

Among sites, the activities of AG, BG, CB and XS were higher in the midstream than those in other sites (Fig. 3a–d), whereas the specific enzyme activities (i.e., activity per unit SOC) showed a little bit different trend with greater level in ZX and BD sites than other sites (Fig. 4a). Among elevations, the activities of four soil enzymes and the specific enzyme activities were generally lower in the low elevation than other elevation zones except for the CB (Table 3; Figs. 4e–h and 4b). Similarly, the activities of four soil enzymes were higher in the top soil than deep soil (Fig. 3i–l), but the specific activity of soil enzymes was no significantly affected by soil depth (Fig. 4c).

### The relationships of SOC and soil enzyme activities with environmental factors

The SOC, LC and C: N ratios of organic pool were significantly positively correlated with plant biomass, plant C and N (Table 4). The RC was positively related to plant C: N ratios, while the LC was negatively correlated with plant C: N ratios. The RIC was positively related to plant N and negatively related to plant C: N ratios (Table 4).

**Table 4:** Pearson correlation coefficients of soil properties on plant traits across different sites and elevations (*P < 0.05; **P < 0.01; ***P < 0.001; numbers are F-values). See Table 1 for the abbreviations.

	Biomass	Plant C	Plant N	Plant C: N ratios
SOC	0.348**	0.331**	0.426**	−0.145
C: N ratios	0.388**	0.376**	0.278**	0.090
LC	0.404**	0.374**	0.508**	−0.281**
RC	0.016	0.036	−0.008	0.217*
RIC	0.207*	0.175	0.290**	−0.332**

The activities of four soil enzymes were significantly positively correlated with SOC, LC, soil temperature (Table 5). The activities of four soil enzymes were significantly negatively correlated with RIC except BG (Table 5). The linear regression analyses further showed significant positive relationships between enzyme activities and soil C contents (Fig. 1S).

**Table 5:** Pearson correlation coefficients of soil enzyme activity on soil properties across different sites, elevations and depths (*P < 0.05; **P < 0.01; ***P < 0.001; numbers are F-values) See Table 1 for the abbreviations.

	SOC	C: N ratios	LC	RC	RIC	pH	SM	ST
AG	0.356**	−0.016	0.415**	0.219**	−0.164*	0.064	−0.124	0.318**
BG	0.394**	0.009	0.398**	0.270**	−0.129	0.015	−0.158	0.377**
CB	0.294**	0.015	0.527**	0.096	−0.302**	−0.206*	−0.278**	0.440**
XS	0.347**	−0.020	0.568**	0.137	−0.308**	−0.117	−0.266**	0.498**

**Figure 5:**
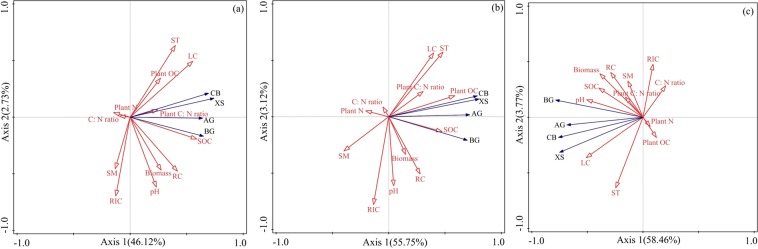
Redundancy Analysis (RDA) results of soil enzyme activities for the soil samples used four soil enzyme activities as species and seven major environmental variables under total samples (**a**); top soil (**b**) and deep soil (**c**). The explanatory variables are shown by different arrows: soil enzyme activities (species) by blue arrows: AG, BG, CB, XS; and environmental variables by the red dashed arrow. See Table 1 for the abbreviations.

The RDA showed that the soil enzyme activities were significantly dependent on environment factors, accounting for 51.49% of the variation (p = 0.002) (Fig. 5a). The first ordination RDA axis (axis 1, horizontal), was highly correlated with SOC and LC, explaining 48.55% of the total variations of the soil enzyme activities (Fig. 5a). The second ordination RDA axis (axis 2, vertical) was mainly related to RIC and explained 2.73% of the total variations of the soil enzyme activities (Fig. 5a). All environment variables presented in the ordination explained 61.74% of the top soil variability of the EAs with the Highest scores (57.45%) on axis 1 being associated with plant C and SOC (Fig. 5b). Additionally, the soil enzyme activities in deep soil were assessed broadly on RDA model, the environmental factors (particularly, the pH and LC) accounted for 61.01% of the variation in the soil enzyme activities (Fig. 5c).

## Discussion

This study revealed spatial variations in soil SOC dynamics and C-hydrolyzing enzyme activities, as well as the linkages between them across the water level fluctuation zone of the Three Gorges Reservoir. These variations could be attributed to indirect effects mediated by plant and direct effects regulated by water flooding. Previous studies have indicated that the reversal of water submergence (i.e. winter flooding) induced by the Three Gorges Dam greatly changes plant species composition and biomass^2,18,39^. Especially, flooding increased C4 (e.g. *Cynodon dactylon*) percentage in the whole water level fluctuation zone, and certainly impact plant C and N content. As we first hypotheses, decreasing trend of SOC content from the upstream to the downstream (Fig. 1a) coincided well with changes in plant biomass and plant C content (Tables 1 and 4), which strongly supported the fact that most SOC exclusively derives from plant residues on site^40^. It has been suggested that soil C-hydrolyzing enzyme activities increase with increasing SOC content^41^. At region scale, the variations in C-hydrolyzing enzyme activities with higher levels in the middle stream compared to other sites (Fig. 3a–d) did not correspond well with the changing trend of SOC (Fig. 1a), but matched with the spatial variation in LC (Fig. 2a).Significantly higher LC content resulted from large new fresh C input (Figs. 2 and 4) could be attributed to higher hydrolytic enzyme activities^28,42^.Moreover, higher C-hydrolyzing enzyme activities in top soil than deep soil (Fig. 3i-l), primarily due to the decreased soil LC with increasing soil depth^32^ (Figs. 2e,g,h). The tightly linkage between C-hydrolyzing enzyme activities and the LC content highlighted the evidence that the LC has been regarded as the pivotal source for microbes to produce hydrolytic enzymes^25,27^, which also supported our second hypothesis. Whereas, the untied linkages between SOC and soil enzyme activities suggested that the enzyme activities were not only affected by SOC concentrations, but also controlled by the SOC turnover and soil C quality (i.e. RIC) in riparian zone^10,23^.

Meanwhile, higher SOC content was found in the middle elevation zone in comparison with other elevation zones (Fig. 1c). As mentioned above, water flooding induced by the Three Gorges Dam altered plant functional type and soil properties^2,32^, and hence plant biomass and C increased by approximately 2-fold in riparian zones (i.e. high and middle elevations) compared to the control during dry season (Table 1), which possibly led to higher SOC contents in middle elevation zone. However, increases in SOC content were not always accompanied by larger plant biomass in high elevation zone (i.e. greater biomass vs. lower SOC content) (Table 1 and Fig. 1c), this phenomenon could attribute to the low sensitivity of SOC to plant changes during anti-seasonal flooding^43–45^. Meanwhile, the SOC content in the low elevation zone without any plant input did not significantly differ with the vegetation coverage zone (high elevation and control zones) (Fig. 1c), possibly due to considerable input of SOC by flooding and leaching into the low elevation zones^5,34^. On the other hand, under continue waterlogging condition in the low elevation zone, the anaerobic environment could hinder soil enzyme activity and hence could lower decomposition rate^46^. This speculation was supported by our results that the significant greater RC content and RIC were observed in the low elevation zone compared to the high elevation zones (Fig. 2e,f), revealing larger recalcitrant fractions with slower decomposition rates^43^. Whereas, the LC showed different changing trend from the SOC and RC, with the lowest level in the low elevation zone^43^ (Fig. 2d), likely due to its faster decomposition rates under flooding zone with lower temperature (Table 2). Therefore, our results revealed plants predominately regulated SOC dynamics possibly by altering litter input, but to some levels, the fluctuating water table in the riparian soil could create alternating aerobic/anaerobic conditions, favoring SOC turnover and sequestration which seemed to overwhelm plant effects in in the riparian zones.

In contrast, all the C-hydrolyzing enzyme activities and the specific enzyme activities were lower in the low elevation zone compared to other elevation zones (Figs. 3e–h and 4b). This variation was possibly because of multiple drives such as changes in soil C content and fraction as well as soil microclimates induced by water flooding^1,43,46^. Indeed, the C-hydrolyzing enzyme activities were positively bound up with the SOC, LC contents, and soil temperature; while parts of enzyme activities were negatively associated with the RIC and soil moisture across sites and elevations (Table 5 and Fig. 5). Interestingly, the changing trend of C-hydrolyzing enzyme activities did not show a consistent trend with the SOC content (Fig. 3e–h vs. Figure 1c), but closely followed variation in LC content along different elevations (Fig. 3e–h vs. Figure 2c). Especially, this finding further confirmed that the LC has been proved to be determinant of enzyme activities in the riparian zones^47–49^ but to some level, disagreeing with previous studies showing tightly positive relationships between enzyme activities and SOC content in terrestrial ecosystems^41,50^. Another explanation for the decreased enzyme activities in the low elevation zone was likely attributed to the low RC and RIC which could hinder microbial activities, and hence reduce C-hydrolyzing enzyme production^25,41^. Meanwhile, the observed lower specific enzyme activities in the low elevation zone could depend on relatively great SOC level (Fig. 1c) and small enzyme activity level (Fig. 3e–h), revealing slow SOC turnover rate in the low elevation zone. Additionally, lower soil temperatures under permanent water submergence (Table 2) in the low elevation zone could strongly affect microbial community composition and function, possibly leading to low enzyme activities^13^.

To conclude, our results demonstrated that soil SOC dynamics and C-hydrolyzing enzyme activities exhibited spatial variations across the water level fluctuation zone of the Three Gorges Reservoir. Variations in C-hydrolyzing enzyme activities were tightly related to soil LC content and fractions, which were affected indirectly by plant composition and directly by water flooding. The SOC, RC contents, and RIC showed decreasing trends from the upstream to the downstream, due to the spatial variation in plant biomass and plant C: N ratios. In contrast, variations in C-hydrolyzing enzyme activities were strongly dependent on LC content and RIC. Meanwhile, the decrease in soil temperatures under permanent water submergence also caused low enzyme activities in the low elevation zone. Moreover, the lower specific enzyme activities in the low elevation zone compared to other elevation zones, indicating slow SOC turnover rate. Thus, our results suggest plants primarily drive SOC dynamics by altering litter input, but to some levels, the roles of water flooding in regulating SOC dynamics seem to overwhelm plant effects in in the riparian zones. However, we should acknowledge the lack of data on soil oxidase activities, and SOC pool derived from a joint contribution fluvial organic matter except plant residues. Thus, to precisely predict the linkages between the SOC dynamics and enzyme activities, we need quantify the potential contribution of fluvial organic matter to SOC, as well as combined with the soil oxidase activities and microbial community compositions in future study.

## Materials and Methods

### Study sites

**Figure 6:**
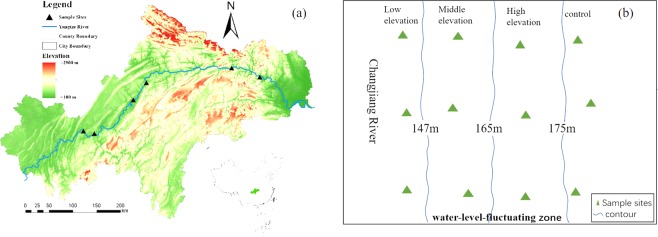
Locations of the sampling sites and different elevations in the water level fluctuation zone of Three Gorges Reservoir, China (**a**) and experimental design (**b**).

In this study, we selected the riparian zone on a large region (29°16′ to 31°25′N, 106° to 111°50′E) from Chongqing to Yichang approximately 660 km in the water level fluctuation of the Three Gorges Reservoir region (forming a total inundation area or reservoir of 1,080 km^2^) (Fig. 6). The climate in this region belongs to subtropical monsoons. The mean temperature is 16.5 °C to 19°C and annual precipitation is approximately 1100mm^18^ with 80% taking place between from April and October. Due to the construction of Three Gorges project, the water level fluctuation zone an area of 349km^2^ is formed with the dry-resetting process opposite to the natural flooding regime^38^. The water level of the reservoir reached to 175 m in winter (October to April) and drop down to 145 m in summer (May to September). The lower zone inundates for 8 almost mouths, the middle zone for 6–7 month, and the higher zone for 3–4 month. The soil involved in our work were predominantly red soil, yellow soil, and mountain yellow soil (Entisols in the World Reference Base). In this study area, The C_3_ plants (e.g. *Bidens pilosa L*.; *Xanthium sibiricum*; *Arundo donax*) predominantly dominated in flooding zone of the upstream (CS, FL and ZX), while the C_4_ (e.g. *Cynodon dactylon*; *Echinochloa crusgalli*) plants dominated in flooding zone of the downstream (WZ, BD and ZG).

### Experimental design and sample collection

Filed investigations were carried out in 6 sites in July- August 2016, when the reservoir’s water level fall in 145 m and the whole riparian zone were exposed to the air after inundation. At each sampling site, four sections of elevation (i.e. low <145 m, inundation area without plant; middle, 145–160 m, longer flooding duration with revegetation; high, 160–175 m, shorter flooding duration with revegetation; and control,>175 m, no-flooding area with original plant) were established, three replicate 1 m × 1 m subplots were selected within each elevation. At each subplot, three soil cores from 0–10 cm and 10–30 cm soil layers were collected at random using a 5 cm diameter, and then mixed three soil cores for one replication in order to reduce sampling errors. A total of 144 soil samples were collected, and soil samples were immediately preserved at −20 °C in fridge and return to the laboratory for biological and biochemical analyses.

Aboveground parts of all plants in each subplot were harvested by scissor in summer (August) for represent the annual aboveground net primary production during the growing season because of anti-season flooding with no carryover of living biomass from previous year^51^, and then oven-dried at 60 °C in the laboratory to a constant weight and weighed.

### Soil properties, soil organic C, LC and RC

Soil pH was measured by a digital pH meter in water at a ratio of 1: 2.5(soil: water). Soil moisture was gravimetrically determined by oven-drying fresh soil at 105 °C for 24 h to a constant weight, and soil temperature was recorded with a temperature probe.

The soil organic C (SOC) contents were measured as previously described by Cheng *et al*.^7^. In briefly, dried soil samples were treated with 1 N HCl at room temperature for 24 h to remove the total inorganic C. Soil recalcitrant C (RC) were obtained by the two-step acid hydrolysis, the first hydrolyzed was add 20 ml of 5 N H_2_SO_4_ to 500 mg soil at 105 °C for 30 min in sealed Pyrex tubes, after cooling the hydrolysate was flushed with de-ionized water and separated by centrifugation and decantation. 2 ml of 26 N H_2_SO_4_ was add into the remaining residue for overnight at room temperature under continuous shaking. Then the acid was diluted to 2 N with deionized water and the residue was hydrolyzed for 3 h at 105 °C with occasional shaking. The residue was washed twice and transferred to centrifuge tube, dried at 60 °C to a constant weight^27,52^. The recalcitrance index for C (RIC) was calculated based on the following equation

$${\rm{RIC}}\,( \% )=({\rm{unhydrolyzed}}\,{\rm{C}}/{\rm{total}}\,{\rm{SOC}})\times 100$$

We selected the dominant species (at least five plants) which accounts for 80% of total biomass in community to illuminate plant C and N content. The sum of dominant species content contribution divided by relative biomass to stand for the community level’s C and N^51^.

The bulk soil and its fractions, and the plant material passed through 100 mesh sieves and then analyzed for C and N content and their isotope composition by an isotope ratio mass spectrometer (Thermo Finnigen, Delta-Plus, Flash, EA, 1112 Series, USA).

### Soil enzyme activities analysis

The enzyme activities involved in α-glucosidase (EC 3.2.1.20) hydrolyzing of soluble saccharides); β-glucosidase (EC 3.2.1.21) hydrolyzing of cellulose; 1,4-β-cellobiosidase (EC 3.2.1.91) hydrolyzing of cellulose; 1,4-β-xylosidase (EC 3.2.1.37) hydrolyzing of hemicellulose, XS), were measured as described by previous studies^32,53^. Briefly, 1 g of field-moist, frozen soil was homogenized by magnetic stirrer in 90 ml of Tris buffer (pH = 7.8) which was approximate pH of the soil samples. The extracellular enzymes were determined by a fluorometric microplate assay using a 100 μM solution of 4-methylumbelliferone (MUB). 50 μL aliquots of 200 μM MUB-substrate were dispensed in the soil assay and control. The slurries were then added to 96- well microplates using eight-channel micropipette for each soil sample, and an additional eight replicates of quench controls. Assays were run with one quenched blank column (50 μL buffer, 100 μL slurries and 50 water), one quenched standard column (50 μL buffer, 100 μL slurries and 50 μL MUB), one MUB-substrate assay column (50 μL buffer, 100 μL slurries and 50 μL MUB-substrate), one blank column (200 μL buffer), one blank standard column (150 μL buffer and 50 μl MUB) one MUB-substrate blank column (150 μL buffer and MUB-substrate). The plates were incubated in the dark for 3 h at 30 °C and measured with a M200 pro (TECEN, Austria) multilabel fluorescence reader using excitation 355 nm and emission 460 nm^54^. Specific enzyme activities were calculated by dividing enzyme activities over the SOC^55^.

### Data analyses

All of the data analyses were performed using SPSS 21.0 software. Multiple-way Analysis of Variance (ANOVA) was used to determine the statistical significance of sites, elevations, soil depths and their interactive effects on parameters including enzyme activities (AG, BG, CB and XS), specific enzyme activities (EA/SOC), soil C (SOC, C: N ratio, LC, RC and RIC), soil properties (pH, SM and ST) and used two-way measures Analysis of Variance (ANOVA) to test the statistical significance of sites and elevation and their interactive effects on plant variables (biomass, plant C, plant N and plant C: N). One-way ANOVA with Tukey’s HSD test was used to determine the statistical significance of sites, elevations and depths on enzyme activities and soil C pools. Pearson correlations analysis was conducted to correlate soil properties on plant traits, as well as soil enzyme activities on soil C and soil properties. The statistical significance of the redundancy Analysis (RDA) was performed using CANOCO software for Windows 4.5 to test the relationship between the soil enzyme activities and environmental variables.
